# Paradigm shift: 'ABC' to 'CAB' for cardiac arrests

**DOI:** 10.1186/1757-7241-18-59

**Published:** 2010-11-15

**Authors:** Umair Khalid, Amyn Abdul Malik Juma

**Affiliations:** 1 Aga Khan University, Medical College, 19/2-A, 32nd Street Off Khayaban-e-Shamsheer, D.H.A., Phase 5, Extension, Karachi, Pakistan

## Abstract

CPR has a proven role in improving survival in cardiac arrest victims, especially those who are outside the hospital. Guidelines published by the AHA have included CPR as a vital intervention for decades. The previous guidelines have focused on the maintenance of airway as the first step, there by delaying the provision of chest compressions. However, the *2010 AHA Guidelines for Cardiopulmonary Resuscitation and Emergency Cardiovascular Care *corrects this by changing the A-B-C of CPR to C-A-B, acknowledging that chest compressions are the most important aspect of the cardiac arrest management.

## 

Cardiovascular disease remains the leading cause of mortality worldwide, with sudden cardiac arrest accounting for approximately half of all these deaths. Since the inception of its first published guidelines by American Heart Association (AHA) in 1966, there have been many updates in the standard treatment protocol for cardiac arrest. However, until recently, the basic formula has remained the same, which involves assessing the conscious state, then checking the Airway, Breathing and Circulation [1-3].

Factors that directly influence the outcome in cardiac arrest include response time of trained health care providers, type of cardiac rhythm on presentation, whether the event was witnessed, and whether the victim received any chest compressions. Regrettably, effective cardiopulmonary resuscitation (CPR) is performed in only 15-30% of these victims. For every minute that CPR and defibrillation is delayed, the chances of survival fall by 7% to 10%. This validates the importance of timely intervention through defibrillation and/or CPR. For out-of-hospital cardiac arrests however, chest compressions remain the mainstay of emergency treatment [1,3].

According to the most recent update being released on 2^nd ^November 2010 [3], there has been paradigm shift towards performing the compressions first, effectively changing the A-B-C of Basic Life Support to C-A-B. These recommendations are based on the most comprehensive resuscitation review ever published, in which 356 resuscitation experts from 29 countries were consulted who reviewed and analyzed the data the 36-month period before the 2010 Consensus Conference.

Other changes in the guidelines [3] are as follows:

1. 'Look, Listen and Feel' has been removed from the BLS algorithm as it was found to be inconsistent and time consuming.

2. Depth of compression for adults has been increased to at least 2 inches and for child to at least 1.5 inches

3. More emphasis on teamwork and training.

The current revisions of guidelines are not without premise. Majority of cardiac arrests occur in adults and the critical element for survival in them is chest compression [4]. In the A-B-C sequence, chest compressions are often delayed due to the complexity of maintaining the airway. It has also been observed that bystanders do not provide CPR most of the times as they find providing rescue breaths the hardest or are not willing to do so. By changing the sequence to C-A-B, chest compressions will be initiated sooner and ventilation minimally delayed. This will presumably improve the survival rates for cardiac arrest in the future.

## Abbreviations

CPR: cardiopulmonary resuscitation; AHA: American Heart Association;

## Competing interests

The authors declare that they have no competing interests.

## Authors' information

UK and AAMJ are recent medical graduates (Class of 2009 & 2010 respectively) of Aga Khan University, Karachi, Pakistan and currently applying for the residency match in United States.

## Authors' contributions

UK conceived the idea after comparing the latest AHA guidelines on the management of cardiac arrest with what was taught to him recently during the AHA certified "Advanced cardiac life support" course, and also drafted the manuscript. AAMJ helped to draft the manuscript and gave final approval to submit manuscript. All authors have read and approved the final manuscript.
